# Beyond the shunt: Divergent insights from hyperoxia testing and contrast echocardiography in adults with pulmonary hypertension

**DOI:** 10.14814/phy2.70594

**Published:** 2025-09-30

**Authors:** A. Beurnier, T. Lacoste‐Palasset, X. Jaïs, L. Savale, A. Bouchachi, M. R. Ghigna, S. Keddache, A. Boucly, F. Bauer, O. Sitbon, M. Humbert, M. Bonay, D. Montani

**Affiliations:** ^1^ Faculty of Medicine Université Paris Saclay Le Kremlin Bicêtre France; ^2^ Inserm UMR_S 999 “Pulmonary Hypertension: Pathophysiology and Novel Therapies” HPPIT, Marie Lannelongue Hospital Le Plessis Robinson France; ^3^ Departement of Physiology–Functional Explorations, DMU 5 Thorinno, bi‐Site Bicêtre Hospital (Le Kremlin Bicêtre) and Ambroise Paré Hospital (Boulogne‐Billancourt) Hospitals Assistance Publique ‐ Hôpitaux de Paris (AP‐HP) Le Kremlin‐Bicêtre France; ^4^ Department of Respiratory and Intensive Care Medicine, Pulmonary Hypertension National Referral Center, FHU André Cournand, ERN‐LUNG, Bicêtre Hospital Assistance Publique ‐ Hôpitaux de Paris (AP‐HP) Le Kremlin‐Bicêtre France; ^5^ Department of Cardiology, Bicêtre Hospital, DMU 4 Correve Assistance Publique ‐ Hôpitaux de Paris (AP‐HP) Le Kremlin‐Bicêtre France; ^6^ Gustave Roussy Department of Biopathology Villejuif France; ^7^ Inserm U1179, END‐ICAP, UFR des Sciences de la Santé‐Simone Veil Université de Versailles Saint‐Quentin‐en‐Yvelines Montigny‐le‐Bretonneux France

**Keywords:** hyperoxia, hypoxemia, pulmonary hypertension, shunt

## Abstract

Our objective was to evaluate the agreement between hyperoxia test (HT) and contrast transthoracic echocardiography (CE) for shunt detection in patients with pulmonary hypertension (PH) and unexplained hypoxemia. We retrospectively collected data from consecutive hypoxemic PH patients over a 2‐year period. The tests were conducted sequentially, and their agreement was assessed using Cohen's kappa coefficient. Fifty‐five PH patients were included, with a median age of 67 years [58–73]; among them, 35 (63.6%) were classified as having pulmonary arterial hypertension (PAH). The mean pulmonary arterial pressure was 39 mmHg [34–49] and pulmonary vascular resistance (PVR) was 5.3 WU [3.8–8.9]. P_A–a_O2 in room air was 50 mmHg [38–61]. A significant shunt was identified by HT (HT+) in 34 patients (61.8%) and by CE (CE+) in 29 patients (52.7%). Discrepancies were observed in 29 cases (52.7%), with a Cohen's kappa index of −0.068. The discordant profiles (HT+/CE− and HT−/CE+) exhibited distinct DLCO *z*‐scores (respectively −3.35 vs. −5.47, *p* = 0.014), while hemodynamic parameters remained similar. The observed lack of agreement may reflect the complementary roles of these tests. Further investigation is needed to explore the potential contribution of micro‐shunts and pulmonary vascular dilations to these findings.

## 1 INTRODUCTION

Pulmonary hypertension (PH) is a haemodynamic condition characterized by the elevation of mean pulmonary arterial pressure (mPAP) greater than 20 mmHg. PH is categorized into five clinical groups based on underlying etiologies. Group 1 corresponds to pulmonary arterial hypertension (PAH), which may be idiopathic, heritable, or associated with conditions such as connective tissue diseases. Group 2 is associated with left heart disease. Group 3 is related to lung diseases and/or hypoxia. Group 4 refers to chronic thromboembolic pulmonary hypertension, and Group 5 includes forms with unclear and/or multifactorial mechanisms (Kovacs et al., 2024). Dyspnea is a cardinal symptom in affected individuals, arising from complex interrelated mechanisms that increase ventilatory drive. Hypoxemia is a frequent finding in these patients, arising from a combination of low mixed venous oxygen tension, decreased cardiac output, diffusion impairment, ventilation/perfusion (VA/Q) mismatching, and/or pathological right‐to‐left shunting (RLS) (Farina et al., 2018; Naeije et al., 2022). RLS is characterized by blood flow bypassing the ventilated areas of the lungs, leading to a significant amount of deoxygenated blood in the systemic arteries with limited improvement under oxygen therapy (Petersson & Glenny, 2014). RLS associated with PH typically occurs in the presence of a patent foramen ovale (PFO), but the implication of arteriovenous malformations has also been described (Shovlin, 2021).

Hyperoxia testing (HT) is used to detect and quantify RLS in clinical practice by revealing an insufficient increase in systemic arterial oxygen pressure under maximal inspired fraction of oxygen (F_i_O_2_ ≃ 1). Contrast transthoracic echocardiography (CE) is another method routinely used for assessing RLS. This test aims to identify an inadequate accumulation of microbubbles in the left heart chambers following their injection into a peripheral vein. In the context of unexplained hypoxemia, intra‐ or extra‐cardiac shunt can be evoked in case of early or delayed visualization of microbubbles during the cardiac cycle, respectively (Bernard et al., 2021; Hampson et al., 2023).

Whether HT and CE provide redundant information in the exploration of hypoxemia among patients with PH remains unclear, due to limited data in the literature. Our objective was to assess the agreement of HT and CE for detecting shunt in routine care among hypoxemic patients with PH.

## 2 METHODS

### 2.1 Population

All patients who underwent routine hyperoxia testing (HT) in the Department of Physiology for the evaluation of suspected shunt between March 15, 2023 (corresponding to the implementation of a standardized procedure for HT with new equipment) and December 31, 2024, were retrospectively screened for inclusion. Patients receiving sotatercept or any investigational drug were not included. Patients were included if they fulfilled all of the following criteria: (1) A confirmed diagnosis of PH according to current international guidelines (Kovacs et al., 2024); (2) Confirmed hypoxemia at the time of HT, defined by alveolar–arterial oxygen tension difference (P_A–a_O_2_) >15 mmHg on room air; (3) CE performed during the same hospital stay.

### 2.2 Blood gas analysis and hyperoxia testing (HT)

As part of routine evaluation in our Center, all patients had two separate radial arterial punctures for blood gas analysis: one performed while breathing room air, and a second one after 30 min of hyperoxia. HT consisted of 30 min of spontaneous ventilation in an upright sitting position while breathing hyperoxic air (F_i_O_2_ = 1), delivered through a face mask equipped with a Hans Rudolph bi‐directional valve connected to an impermeable balloon (COSMED, Italy). ABG samples were analyzed using the ABL90 analyzer (Radiometer Medical ApS, Denmark). As suggested by other authors, a shunt was assumed if the P_a_O_2_ did not reach 500 mmHg (Nanthakumar et al., 2001). P_A–a_O_2_ was estimated as 713 × F_i_O_2_ − P_a_CO_2_/0.8 − P_a_O_2_ where F_i_O_2_ = 0.21 in room air and F_i_O_2_ = 1 during hyperoxia testing. Percentage of shunting (Qs/Qt) was estimated using Chiang's formula (Chiang, 1968) based on P_A–a_O_2_ in 100% FiO2 (P_A–a_O_2 100%_) as follow: P_A–a_O_2 100%_/(P_A–a_O_2 100%_ + 5/0.003).

### 2.3 Contrast transthoracic echocardiography (CE)

The CE procedure was performed and interpreted by a constant group of trained echocardiographers, following a locally standardized procedure based on standard practice reports (Bernard et al., 2021; Senior et al., 2009; Silvestry et al., 2015). Each operator provided one of the following conclusions: absence of shunt, CE consistent with intracardiac shunt, or extracardiac shunt. As part of this real‐world study, some operator‐dependent methodological variations were accepted (e.g., position changes, bubble reinjection). When contrast echocardiography did not demonstrate spontaneous bubble passage, a Valsalva maneuver was systematically performed. In cases where spontaneous passage was observed, the decision to reinject bubbles while performing a Valsalva maneuver was left to the operator's discretion in this real‐world setting. In most cases, patients were first positioned in the left lateral position; microbubbles were created by agitating a mixture of 9 mL of saline solution and 1 mL of air and injected within 3 s in an upper limb vein. The potential appearance, timing, and origin of microbubbles in the left‐sided heart chambers were closely monitored in the apical four‐chamber view over at least 20 cardiac cycles. A bubble passage within < 3 cycles being classified as early and a passage at four or more cardiac cycles was classified as late. The number of microbubbles was quantified for each subject, in order to provide a semi‐quantitative evaluation of contrast on a scale from 0 to 3 as described elsewhere (Velthuis, Buscarini, et al., 2015).

### 2.4 Ethics statement‐data collection and analysis

In France, retrospective analyses of anonymized data do not require specific Institutional Review Board approval or additional informed consent. This study complied with the Declaration of Helsinki (1975, revised in 2008 and 2024). Data were collected from the French Pulmonary Hypertension Registry (https://registre‐htap.aphp.fr), which operates under the approval of the Commission Nationale Informatique et Libertés (CNIL, approval number 842063, May 24, 2003). Use of registry data for research purposes was approved by the Scientific Committee of the French PH Registry. All data were anonymized in accordance with CNIL requirements. The patient was individually informed both at registry inclusion and during hospital care about the potential research use of his medical data, including possible publication of anonymised images, and was offered the possibility to object. No objection was recorded. This approach is fully compliant with the French data‐protection framework (GDPR + Loi “Informatique et Libertés”) and the CNIL MR‐004/MR‐003 reference methodologies, which require prior information and a right to object for such studies. Anthropometric characteristics, hemodynamics data, and carbon monoxide diffusion variables during the same hospital stay were collected. In the event of unavailability of these variables during this period, the closest time‐point possible value was selected. Diffusion capacity of the lungs for carbon monoxide (DLCO) and transfer coefficient of the lung for carbon monoxide (KCO) were expressed as *z*‐scores and percent of predicted value using GLI equations after correction for hemoglobin level (Stanojevic et al., 2017). Each DLCO maneuver and quality grading were carefully reviewed to ensure that the chosen value was in accordance with current guidelines (Graham et al., 2017). Specifically, the DLCO value was determined as the average of two or more grade A maneuvers meeting the repeatability criterion. If only one grade A maneuver was obtained, the DLCO value from that maneuver was used. In the absence of grade A maneuvers, the average of grade B–D maneuvers was taken. Maneuvers graded *F* were deemed unusable. Values across groups were compared using the Kruskal–Wallis test. Pairwise comparisons were then performed using Dunn's procedure with a Bonferroni correction to adjust for multiple testing. Agreement between CE and hyperoxia testing was assessed using Cohen's kappa statistic (*κ*).

## 3 RESULTS

### 3.1 Characteristics of the population

Fifty‐five patients were included. Detailed characteristics of the population are provided in Table 1. The median age was 67.3 years [57.9–72.6] and the P_A–a_O_2_ in room air was 49.6 mmHg [38.3–61.1]. The median mPAP was 39.0 mmHg [33.5–49.0], PVR was 5.3 [3.8–8.9], and CI was 2.8 [2.4–3.4]. Thirty‐five (63.6%) were categorized as PAH (group 1). Among PAH patients, 17 were diagnosed with porto‐pulmonary arterial hypertension (PoPH), 7 with PAH and overt features of venous/capillary involvement (PVOD), and 7 with idiopathic PAH. The remaining PAH patients had congenital heart disease (*n* = 1), heritable PAH with no identifiable pathogenic mutation (*n* = 1), scleroderma (*n* = 1), and HIV infection (*n* = 1). Reliable DLCO values were obtained for 50 patients (45 with at least one grade A measurement and 5 with grade B and/or C measurements) since 5 were unable to complete the DLCO testing procedure adequately.

**TABLE 1:** Characteristics of patients.

	All, *n* = 55	Group 1, *n* = 35	Group 3, *n* = 6	Group 4, *n* = 9	Group 2 and 5, *n* = 5
Age (years)	67.3 [57.9–72.6]	66.7 [57.6–71.8]	71.2 [67.2–77.4]	69.8 [63.3–78.4]	69.5 [67.7–69.6]
BMI (kg m^−2^)	28.3 [24.5–33.4]	28.3 [25.2–33.0]	23.8 [22.4–25.6]	32.0 [23.7–33.1]	37.1 [33.6–39.3]
Male/Female	28/27	18/17	6/0	2/7	3/2
Basal P_a_O_2_ (mmHg)	58.6 [47.9–68.8]	59.8 [48.1–74.7]	49.0 [43.2–50.3]	62.0 [60.4–65.1]	48.6 [43.0–52.1]
Basal P_A‐a_O_2_ (mmHg)	49.6 [38.3–61.1]	49.2 [34.8–60.0]	64.1 [61.0–66.5]	40.6 [38.4–48.6]	53.2 [48.3–57.9]
DLCO (% pred)	45.7 [32.8–68.1]	45.0 [37.3–65.0]	24.5 [20.9–32.6]	68.2 [48.1–76.1]	22.1 [20.9–69.4]
DLCO (*z*‐score)	−3.9 [−2.1 to −5.0]	−3.7 [−2.4 to −4.8]	−6.1 [−6.7–4.9]	−2.1 [−3.5 to −1.6]	−6.5 [−7.4 to −1.9]
KCO (% pred)	59.5 [37.5–78.6]	62.5 [44.9–77.5]	29.3 [26.6–35.7]	81.1 [61.7–90.3]	31.4 [31.2–73.8]
KCO (*z*‐score)	−2.6 [−4.2 to −1.4]	−2.6 [−3.9 to −1.5]	−5.0 [−5.3 to −4.3]	−1.2 [−2.5 to −0.6]	−4.9 [−5.8 to −1.6]
mPAP (mmHg)	39.0 [33.5–49.0]	39.0 [33.5–47.0]	46.0 [38.0–57.8]	50.0 [40.0–54.0]	37.0 [32.0–37.0]
CI (L/min/m^2^)	2.8 [2.4–3.4]	2.9 [2.4–3.7]	2.6 [2.3–2.7]	2.7 [2.6–3.0]	2.8 [2.2–2.8]
PVR (WU)	5.3 [3.8–8.9]	5.2 [3.7–7.9]	8.4 [5.9–8.9]	5.8 [4.5–9.7]	4.9 [2.4–7.9]

*Note*: Quantitative data were expressed as median [interquartile range].

Abbreviations: BMI, body mass index; CI, cardiac index; DLCO, diffusion capacity of the lungs for carbon monoxide; KCO, Transfer coefficient of the lung for carbon monoxide; mPAP, mean pulmonary arterial pressure; NA, not applicable; P_a_O_2_, partial pressure of oxygen in arterial blood; P_A‐a_O_2_, alveolar–arterial tension difference in oxygen; PVR, pulmonary vascular resistance.

### 3.2 Identification of shunting and concordance between tests

**FIGURE 1:**
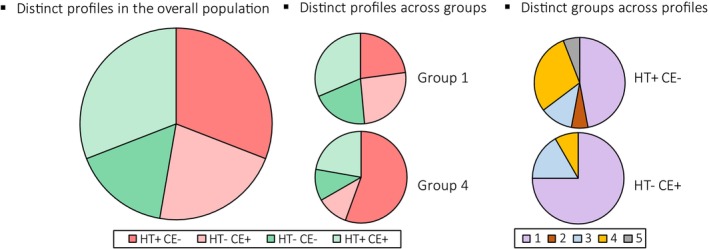
Concordance of tests in detecting shunts in hypoxemic patient with pulmonary hypertension. CE, contrast transthoracic echocardiography; HT, hyperoxia testing; + indicates an abnormal test (bubble passage or insufficient increase in arterial oxygen pressure); − indicates a normal test (no bubble passage or arterial oxygen tension increase above 500 mmHg). Left panel: concordance in the overall population. Middle panel: concordance within the two largest PH group. Right panel: distribution of each PH group in the two discordant profiles.

HT indicated an abnormal shunt (HT+) in 34 patients (61.8%) with a median P_a_O_2_ in hyperoxia of 400.0 mmHg [321.8–446.0], which corresponded to an estimated shunting of 13.6% [11.5–17.2]. CE was suggestive of abnormal shunt (CE+) in 29 patients (52.7%), mostly characterized by a late passage of bubbles (21/29). CE revealed grade 1 bubble passage in 5 patients, grade 2 in 15 patients, and grade 3 in 9 patients. Discrepancies in shunt detection were observed in 29 cases (52.7%). The Cohen's kappa index was −0.068, indicating disagreement between tests. All four possible outcomes of the two tests were observed: HT+/CE+ (*n* = 17), HT−/CE− (*n* = 9), HT+/CE− (*n* = 17) and HT−/CE+ (*n* = 12), with a distinct profile across PH groups and distinct PH groups associated with each discordant profile (Figure 1).

### 3.3 Characteristics of patients with discordant outcomes

**FIGURE 2:**
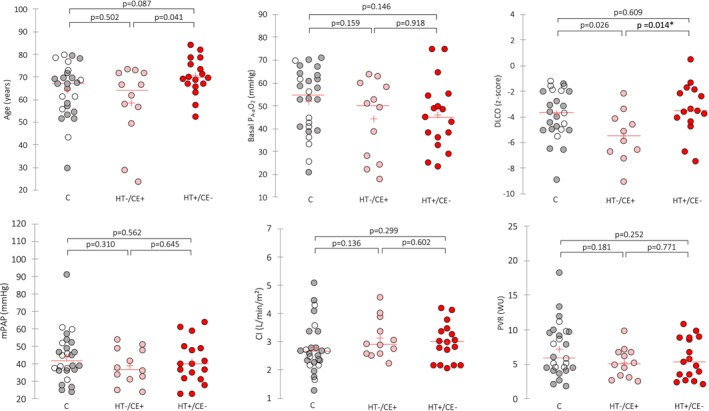
Comparison between each discordant profile and the concordant one. *p‐value below the significance threshold after Bonferroni correction. + indicates an abnormal test (bubble passage or insufficient increase in arterial oxygen tension); − indicates a normal test (no bubble passage or an arterial oxygen pressure increase above 500 mmHg). CE, bubble‐enhanced transthoracic echocardiography; C, concordant profiles, empty circles corresponding to HT−/CE‐ and filled circles to HT+/CE+. CI, cardiac index; DLCO, diffusion capacity of the lungs for carbon monoxide; HT, hyperoxia testing; mPAP, mean pulmonary arterial pressure; P_A‐a_O_2_, alveolar–arterial tension difference in oxygen; PVR, pulmonary vascular resistance.

When comparing each discordant profile (HT+/CE− and HT−/CE+) with the concordant one (HT+/CE+ or HT−/CE−), DLCO z‐score was found to be significantly different across the three groups (Kruskal–Wallis statistics = 6.685, *p* = 0.035). Post‐hoc analysis found lower DLCO z‐scores among HT−/CE+ as compared with HT+/CE− (−5.47 vs. −3.35, *p* = 0.014) with a trend towards lower age (58.6 vs. 70.4 years, *p* = 0.041). Late passage of bubbles was observed in 10/12 patients of the HT−/CE+ group (early intense opacification consistent with PFO in 2 other cases). No differences were observed regarding basal hypoxemia and hemodynamic parameters across the groups (Figure 2).

### 3.4 Illustrative case‐report and pathologic correlation in a patient with discordance HT−/CE+ results

**FIGURE 3:**
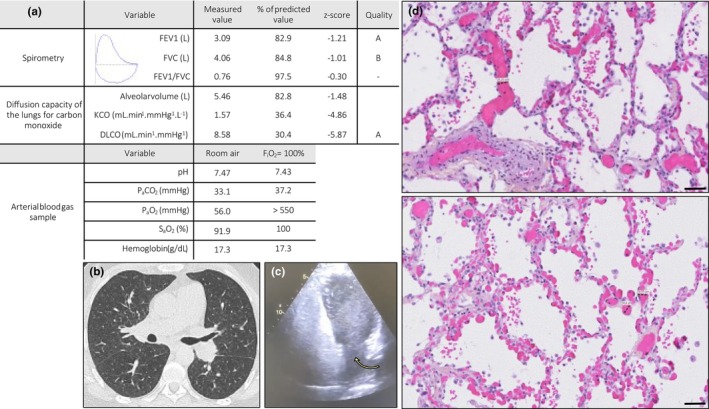
Investigation of severe hypoxemia in a patient with pulmonary arterial hypertension. (a) Spirometry results were normal and DLCO was severely impaired, with a normal response to hyperoxia. (b) The chest CT does not reveal any significant parenchymal abnormalities. (c) The contrast transthoracic echocardiography showed a late and massive passage of bubbles at the 6th cycle originating from the pulmonary veins (yellow arrow). (d) The histopathological analysis reveals marked pulmonary vascular dilations explaining the bubble passage, among pulmonary haemangiomatosis foci (scale bar = 50 μm). The close continuity between those vessels and the alveoli walls is consistent with the normal response to hyperoxia (absence of true shunt). Impaired gas diffusion due to vascular malformations may contribute significantly to the patient hypoxemia. DLCO, diffusion capacity of the lungs for carbon monoxide; FEV1, forced expiratory volume in 1 s; FVC, forced vital capacity; KCO, transfer coefficient of the lung for carbon monoxide; P_a_CO_2_, systemic arterial partial pressure of carbon dioxide; P_a_O_2_, systemic arterial partial pressure of oxygen; S_a_O_2_, systemic arterial oxygen saturation of hemoglobin.

We report the case of a patient in his fifties from the HT−/CE+ group, referred for suspected PH (Figure 3). He had been followed for several years by a pulmonologist for a presumptive diagnosis of COPD, despite normal spirometry and no smoking history. Long‐term oxygen therapy had been initiated previously, and later worsening hypoxemia prompted referral to a secondary hospital. Echocardiography showed an intermediate probability of PH. Computed tomography (CT) pulmonary angiography and ventilation‐perfusion lung scan excluded chronic thromboembolic disease and significant parenchymal involvement.

Right‐heart catheterization confirmed severe precapillary PH (PVR = 5 WU). ABG on room air revealed severe hypoxemia (P_a_O_2_ = 56 mmHg). Pulmonary function tests showed normal spirometry but severe impairment of the diffusing capacity for carbon monoxide (DLCO) at 30% of predicted values. CE demonstrated delayed and massive bubble appearance from the 6th cardiac cycle, whereas the hyperoxia test showed a normal response (PaO2 >550 mmHg). A diagnosis of PAH with features of venous/capillary involvement (PVOD/PCH) was suspected, and a cautious therapeutic strategy with tadalafil monotherapy was initiated. No hemodynamic improvement was observed during follow‐up. Due to clinical deterioration and diagnosis of PVOD, the patient was listed for and successfully underwent bilateral lung transplantation.

Pathological assessment of the explant lungs confirmed the diagnosis of PVOD and revealed multiple areas of dilated peripheral pulmonary vessels in close proximity to alveolar structures. This pathological pattern may provide a physiological explanation for the observed combination of delayed bubble passage, severely impaired DLCO, and preserved response to hyperoxia.

## 4 DISCUSSION

This study demonstrates the disagreement between CE and HT for detecting a shunt in hypoxemic PH patients assessed in an expert center during routine clinical practice, suggesting distinct mechanisms underlying the hypoxemia in those patients. In 12 patients (22%), bubble passage was associated with a normal response to hyperoxia (HT−/CE+). This particular profile has also been observed in patients with hereditary hemorrhagic telangiectasia (Velthuis, Vorselaars, et al., 2015). In our cohort of PH patients, we suggest that the presence of pulmonary vascular dilations is a plausible explanation for this finding. Indeed, thin‐walled vein‐like dilated vessels have been previously described distally to plexiform lesions in PAH patients (Pietra et al., 2004) and capillary dilation is a recognized feature of capillary bed alteration in PVOD (Ghigna & Dorfmüller, 2019). We can hypothesize that the enlarged vessels would allow the passage of microbubbles, while their close continuity with the alveolar walls could enable an appropriate response to hyperoxia, thus inducing hypoxemia through mechanisms distinct from true shunting. In the present study, we provide three observations that support this hypothesis: first, the late passage of bubbles in almost all those cases is consistent with intrapulmonary transit through vascular dilations. Second, this group of patients exhibited significantly lower values of DLCO; although such differences should be interpreted with caution–since DLCO not only reflects intrinsic gas exchange properties, but also lung size and ventilation distribution– this finding may be anticipated in the context of capillary dilation, which can disrupt normal diffusion dynamics (Michael & Hughes, 2021). Third, we were able to visualize pulmonary dilations on the histopathological analysis of an explant from a patient in that group (the only patient of the cohort who benefited from lung transplantation during the study period). Taken together, these findings highlight that CE and HT are more complementary than redundant, as they could reveal specific mechanisms of hypoxemia in PH patients. Our results gain further relevance in light of recently published case reports. Olsson et al. (2025) reported severe hypoxemia in two patients treated with sotatercept, a novel therapeutic option for PAH. Both cases were characterized by normal HT and positive CE with late passage of bubbles, attributed to pulmonary capillary dilatations. In line with our findings, those profiles (HT−/CE+) were associated with low values of DLCO. These observations corroborate those of Mitchell et al. (2025) who described new‐onset intrapulmonary transit of bubbles in 3 PAH patients treated with sotatercept. Within our series, three patients with idiopathic or heritable PAH exhibited late bubble passage; none were receiving sotatercept, and one had a normal hyperoxia test. PAH treatments are frequently associated with a decrease in arterial oxyhemoglobin saturation, which has been linked to reduced survival (Valentin et al., 2021): clarifying the impact of hypoxemia associated with vascular dilations on therapeutic response and long‐term outcomes would be highly valuable.

Seventeen patients (31%) in our cohort had abnormal HT results indicative of a shunt, despite the absence of any visible bubble passage on CE. This observation has been previously reported by Vodoz et al. (2009): in a cohort of 34 PH patients with shunt diagnosis based on HT, the authors did not find evidence of bubble passage in 28 of these patients. We can hypothesize that some patients develop intrapulmonary micro‐shunts that are significant enough to induce hypoxemia but too small in size to allow the passage of bubbles. The involvement of intrapulmonary shunts associated with micro‐vasculopathy may be a contributing factor to hypoxemia in chronic thromboembolic pulmonary hypertension (CTEPH) (Minatsuki et al., 2019) (Reimann et al., 2023). Intrapulmonary shunting has been involved in the hypoxemia of COPD patients with severe PH, along with greater VA/Q mismatch and reduced venous oxygen tension (Piccari et al., 2023). Intrapulmonary bronchopulmonary anastomoses have also been described in idiopathic PAH (Galambos et al., 2016), but the clinical implication of such pathophysiological events remains uncertain.

The following objections can be raised to contest the observed discordance. First, we cannot exclude the possibility of false negatives in CE because this test is influenced by the patient's position and the operator's technique. While the potential inter‐operator variability limits the mechanistic interpretation of our findings, the real‐world setting of our study enhances the generalizability of the conclusion regarding the discordance between the tests in clinical practice. Second, the causal relationship between bubble passage and the underlying hypoxemia can be questioned, especially given that a persistent PFO can be found in 38% of healthy, young, asymptomatic subjects (Elliott et al., 2013). Conducting a quantitative contrast assessment in a prospective study would be useful for better understanding this issue. Notably, in our cohort, a PFO was diagnosed in eight patients: its association with an abnormal response to hyperoxia in six cases supported right‐to‐left shunting through the PFO as the cause of hypoxemia. Transient stagnation of blood in the pulmonary veins during the strain phase after a Valsalva maneuver can also lead to pseudo contrast and false positivity of CE (Soliman et al., 2007). However, in our study, all delayed bubble passages were observed spontaneously, which reasonably rules out this bias. Thirdly, we cannot exclude false positive HT. Indeed, the development of absorption atelectasis leading to intrapulmonary shunting under hyperoxic conditions is plausible, as this phenomenon has been documented during preoxygenation in the induction of general anesthesia. (Bignami et al., 2019). Although lung ultrasound might be an interesting tool to monitor that phenomenon (Kim et al., 2020), this bias is difficult to mitigate in routine clinical practice. Based on animal models, we also could speculate that a mechanism of pre‐ or non‐capillary oxygenation may contribute to falsely normal results in the HT. Notably, this mechanism is thought to be impaired in PH (Tabuchi et al., 2013). Forth, defining the shunt according to the estimated Qs/Qt ratio may be advantageous, but the corresponding threshold is subject to debate, with some authors using 5% (Vodoz et al., 2009), while others consider 10% to be more clinically relevant (Carta et al., 2022) (Reimann et al., 2023). In our study, using a 7.5% cutoff for the hyperoxia test would reclassify only five patients, without altering our conclusions. It should be noted that Qs/Qt estimation with Chiang's formula (Chiang, 1968) relies on several assumptions that may reduce its applicability and relevance in the specific setting of PH patients. Notably, the assumed arteriovenous oxygen content difference of 5 mL% is most likely underestimated among patients with cardiocirculatory failure (Nirmalan et al., 1998). Finally, an impact of hyperoxia itself on bubble passage in PH patients may be debated because, in healthy subjects, it has been shown that hyperoxia can prevent intrapulmonary transit of bubbles during exercise (Lovering et al., 2008). In PH patients, hyperoxia has been proved to decrease PVR independently of the Qs/Qt ratio (Carta et al., 2022). Importantly, CE and HT were never performed simultaneously in our cohort. The relatively advanced median age of our cohort also warrants caution when extrapolating these findings to young patients with PH.

In conclusion, our findings indicate that contrast transthoracic echocardiography and hyperoxia testing offer complementary insights for the assessment of hypoxemia in PH patients, potentially enabling the identification of distinct phenotypes. (1) A positive contrast transthoracic echocardiography result does not always correspond to the presence of shunting, possibly due to pulmonary vascular dilations that compromise gas exchange through alternative mechanisms such as diffusion impairment. (2) An abnormal hyperoxia test is not necessarily associated with visible bubble passage: the role of intrapulmonary micro‐shunts requires further investigation.

## FUNDING INFORMATION

No funding was received for this study.

## CONFLICT OF INTEREST STATEMENT

A. Boucly reports personal fees from Astrazaneca, AOP Orphan, Ferrer, Gossamer, Janssen, MSD, United Therapeutics. M. Humbert reports grants from Gossamer, Merk, personal fees from 35 Pharma, Aerovate, AOP Orphan, Chiesi, Ferrer, Gossamer, Inhibikase, Janssen, Johnson & Johnson, Keros, Liquidia, Merck, Novartis, Regeneron, Respira, Roivant, United Therapeutics. X. Jaïs reports grants from Acceleron, Janssen, MSD, Bayer Healthcare, personal fees from Janssen, MSD. T. Lacoste‐Palasset reports research grants from La Fondation Maladies Rares and financial support from Oxyvie. D. Montani reports grants from Gossamer, Merk, personal fees from Ferrer, Merck, Bayer, Boehringer, Chiesi, GSK, Jazz Pharmaceuticals. L. Savale reports grants from Acceleron, Janssen, Merk and personal fees from Bayer, Janssen, Merk. O. Sitbon reports grants from AOP Orphan, Ferrer, Gossamer, Janssen, MSD, personal fees from Altavant/Enzyvant, AOP Orphan, Ferrer, Gossamer Bio, Janssen, Liquidia, MSD, Pulmovant, Respira Therapeutics, United Therapeutics. The remaining authors declare that they have no conflicts of interest to disclose.

## Data Availability

Datasets are available from the corresponding author upon reasonable request.
